# Increasing the Dose and/or Repeating Faecal Microbiota Transplantation (FMT) Increases the Response in Patients with Irritable Bowel Syndrome (IBS)

**DOI:** 10.3390/nu11061415

**Published:** 2019-06-24

**Authors:** Magdy El-Salhy, Trygve Hausken, Jan Gunnar Hatlebakk

**Affiliations:** 1 Section for Gastroenterology, Department of Medicine, Stord Hospital, Box 4000, 54 09 Stord, Norway; 2 Department of Clinical Medicine, University of Bergen, 5007 Bergen, Norway; trygve.hausken@helse-bergen.no (T.H.); jan.gunnar.hatlebakk@helse-bergen.no (J.G.H.); 3 National Centre for Functional Gastrointestinal Disorders, 5021 Bergen, Norway

**Keywords:** abdominal symptoms, dysbiosis, faecal microbiota transplantation, fatigue, IBS, quality of life

## Abstract

Background: Faecal microbiome transplantation (FMT) appears to be an effective method for treating irritable bowel syndrome (IBS) patients. However, it is not clear if a high transplant dose and/or repeating FMT are/is needed to ensure a response. The present study was undertaken to clarify this matter. Methods: Ten IBS patients who did not respond to a 30-g transplant subsequently received a 60-g transplant into the duodenum via a gastroscope. The patients provided faecal samples before and 1 month after FMT. They completed five questionnaires measuring symptoms, fatigue and quality of life at baseline and then at 2 weeks, 1 month and 3 months after FMT. The dysbiosis index (DI) was measured using the GA-map Dysbiosis Test^®^. Results: Seven patients (70%) responded to the 60-g transplant, with significant clinical improvements in the abdominal symptoms, fatigue and quality of life in 57%, 80% and 67% of these patients. The 60-g transplant also reduced the DI. Conclusion: FMT is an effective treatment for IBS. A high-dose transplant and/or repeated FMT increase the response rate and the intensity of the effects of FMT.

## 1. Introduction

Irritable bowel syndrome (IBS) is a chronic gastrointestinal disorder affecting 12.1% of the world population [1,2]. There are no biochemical or radiological tests for the diagnosis of IBS, and the diagnosis of IBS is based on symptom criteria such as the Rome criteria [3]. IBS affects the patients’ quality of life in the same degree as major chronic diseases such as diabetes, liver and kidney failure [3]. There is no effective treatment for the condition, and the current treatment in the clinic is symptomatic [3].

The aetiology of IBS appears to involve multiple factors, including genetics, environment, social learning, dietary intake, intestinal microbiota, low-grade inflammation and disturbances in the endocrine cells of the gastrointestinal tract [1]. The intestinal bacterial composition differs between IBS patients and healthy subjects [4,5,6,7,8,9,10,11,12,13,14,15,16,17]. The role of the intestinal microbiome in several diseases and disorders including IBS has received considerable attention recently [15,16,17,18]. The composition and proportions of microbial fractions in healthy subjects are affected by several factors, including genetic factors, diet, social behaviours and other environmental factors [18]. The ideal microbiome composition and bacteria species that are important in the pathophysiology of IBS remain unclear [15,18,19]. Moreover, the impact of low diversity of intestinal bacteria (dysbiosis) in IBS patients is still debated [16,17,18].

Faecal microbiome transplantation (FMT) has been performed in patients with IBS with the aim of restoring their intestinal microbiota to a healthy condition [18,20,21]. Applying FMT to a small cohort of IBS patients in open-label studies produced positive results [18,20,21]. However, conflicting results have been reported for two recently published randomised double-blind placebo-controlled studies [22,23].

The outcome of FMT seems to be donor-dependent, and the utilization of a so-called superdonor appears to be essential for successful FMT [24]. A superdonor has been defined as a healthy subject who is normobiotic and has a favourable bacterial signature [24]. We recently conducted a randomised, double-blind placebo-controlled study using a single superdonor, and found that the response rate was higher and the effect was stronger in patients who received 60-g transplants than in those who received 30-g transplants [25]. The present study was conducted to establish whether the non-responders to a 30-g transplant in the previous study would respond to a 60-g transplant.

## 2. Material and Methods

### 2.1. Study Design

Patients who did not respond to a 30-g transplant in the previous randomised double-blind placebo-controlled study were enrolled in the present open-label study to receive a 60-g transplant.

The patients provided a faecal sample at the baseline and completed five questionnaires to measure their symptoms, fatigue and quality of life. The patients provided another faecal sample at 1 month after FMT, and they completed a further set of questionnaires at 2 weeks, 1 month and 3 months after FMT, which were sent by post. Polyethylene glycol and loperamide were allowed during the intervention as rescue medication.

### 2.2. The Donor

The donor used has been described in detail elsewhere [25]. In summary, he was a 36-year-old male with a body mass index (BMI) of 23.5 kg/m^2^. He was non-smoker and did not take any medication. He was born by vaginal delivery and breastfed. He was screened according to the standard guidelines for FMT donors [26]. In summary, he was interviewed and his medical history and lifestyle habits were reviewed to exclude any exposure to infectious agents or risky social or sexual behaviour such as drug or alcohol abuse. In addition, gastrointestinal, metabolic, and neurological disorders were excluded through physical examination and blood tests. He had also undergone serological tests and stool cultures/examinations in order to avoid any risk of transmitting an infectious disease to the recipients.

The donor regularly consumed dietary supplements rich in protein, vitamins, fibre and minerals. Before using his faeces, an analysis of his faecal bacteria showed a dysbiosis index (DI) of 1, indicating normobiosis. The donor bacterial profile deviated from the normal abundance in 14 out of the 39 bacteria markers tested. Twelve of the deviating bacteria belonged to phylum Firmicutes, one in the phylum Proteobacteria and one in the phylum Verrucomicrobia. Of the bacteria belonging to the Firmicutes phylum, *Lactobacillus*, *Streptococcus* and *Dorea *spp. were higher than the normal levels.

### 2.3. Patients

Ten IBS patients who did not respond to a 30-g transplant in the previous study [24] were included in the present study, and received a 60-g transplant at 3–4 months after the first transplant. They had IBS according to the Rome IV criteria without alarming symptoms. The medical history was obtained for all of the patients, and they were examined physically. In addition, they were examined by gastroscopy and colonoscopy to exclude other gastrointestinal diseases. All of the patients adhered to a modified NICE (National Institute for Health and Care Excellence) diet. They comprised eight females and two males aged 39.1 ± 9.0 years (mean ± SD), and three of them had diarrhoea-dominated IBS (IBS-D), two had constipation-dominated IBS (IBS-C) and five had mixed IBS (IBS-M).

### 2.4. Collection, Preparation and Administration of Faecal Samples

All faecal samples were frozen immediately and kept at −20 °C until they were delivered frozen to hospital on the same day, where they were kept at −80 °C. The faecal samples of the donor were thawed at 4 °C for 2 days before FMT. On the day of the FMT, the faecal samples were weighed, and each 30 g of the faeces was mixed with 40 mL of sterile saline, filtered through a 110 × 10 cm non-woven swab (One Med, Helsinki, Finland), drawn into 50-mL sterile syringes, sealed and kept at 4 °C until the time of the FMT. To achieve a 60-g transplant, 80 mL of this solution was injected through the working channel of a gastroscope into the distal duodenum. The working channel of the gastroscope was then flushed with another 40 mL of sterile saline.

### 2.5. Questionnaires

Abdominal symptoms were assessed by the IBS Symptom Severity Score (IBS-SSS) and Birmingham IBS symptom questionnaires [27,28]. Some of the items of these two questionnaires overlap, but they were both completed since the IBS-SSS lacks items about diarrhoea and constipation, and the Birmingham IBS symptom questionnaire does not contain an item about abdominal distension. Fatigue was measured using the Fatigue Assessment Scale (FAS) [29]. Quality of life was measured by the IBS Quality of Life (IBS-QoL) and the Short-Form Dyspepsia Index (SF-NDI) questionnaires [30,31,32]. These questionnaires are complementary since the SF-NDI contains a specific item about work/study that the IBS-QoL questionnaire lacks. In contrast to the IBS-QoL, which measures the quality of life in such a way that an increase in the score indicates an improvement in the quality of life, the SF-NDI measures the reduction in the quality of life, with a decrease in the score indicating an improvement in the quality of life.

A reduction of the total IBS-SSS of ≥50 points after the FMT was considered a response. Furthermore, significant clinical improvements in abdominal symptoms, fatigue and quality of life were considered to have occurred in patients with a decrease of ≥175 points in the total IBS-SSS, a decrease of ≥4 points in the FAS score and an increase of ≥14 points in the IBS-QoL score, respectively [27,31,33].

### 2.6. Dysbiosis Index

The DI was measured using a commercially available test (GA-map Dysbiosis Test^®^, Genetic Analysis, Oslo, Norway). The method has been described in detail elsewhere [34,35].

### 2.7. Ethics

This study was approved by the Regional Committee for Medical and Health Research Ethics West, Bergen, Norway (approval no. 2017/1197/REK vest). All subjects provided oral and written consent to participate. The study was registered at www.clinicaltrials.gov (NCT03822299).

### 2.8. Statistical Analysis

The paired *t*-test and Kruskal–Wallis test with Dunn’s multiple-comparisons test as a post-test were used as applicable. A probability value of *p* < 0.05 was considered significant.

## 3. Results

Seven of the ten patients responded to the 60-g transplant (70%). The three non-responders belonged to the IBS subtypes IBS-D (*n* = 1), IBS-C (*n* = 1) and IBS-M (*n* = 1).

### 3.1. Questionnaires

**Figure 1:**
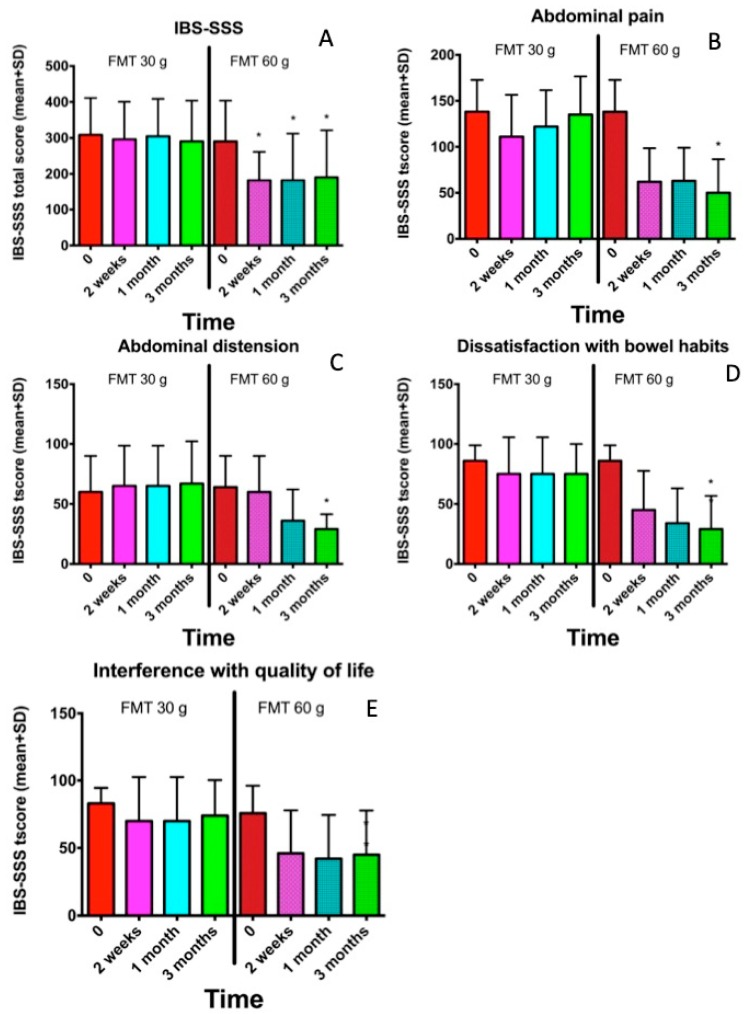
The Irritable Bowel Syndrome Symptom Severity Score (IBS-SSS) at different intervals after receiving 30-g and 60-g transplants for the total score (**A**), abdominal pain (**B**), abdominal distension (**C**), dissatisfaction with bowel habits (**D**) and interference with quality of life (**E**). * *p* < 0.05 compared to baseline. FMT: faecal microbiome transplantation.

**Figure 2:**
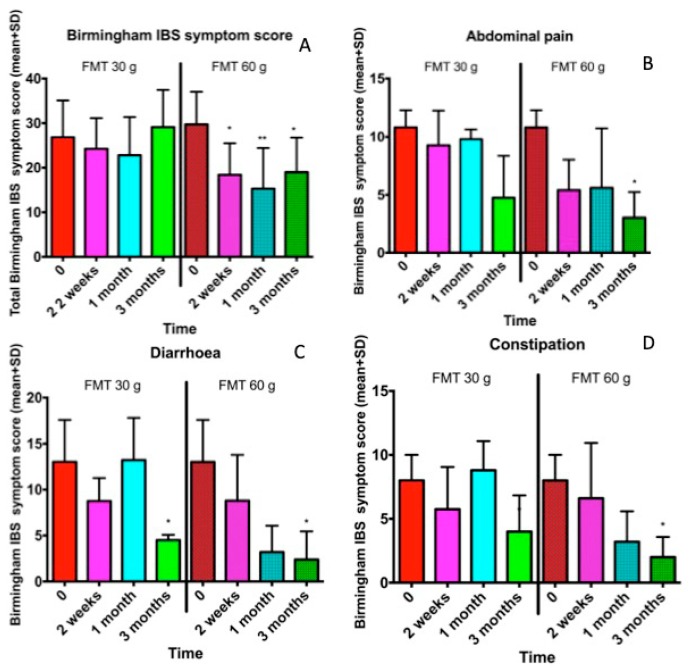
The total Birmingham IBS symptom score and its three items in IBS patients who initially received a 30-g transplant that was subsequently followed by a 60-g transplant for the total score (**A**), abdominal pain (**B**), diarrhoea (**C**) and constipation (**D**). * *p* < 0.05; ** *p* < 0.01 compared to baseline.

**Figure 3:**
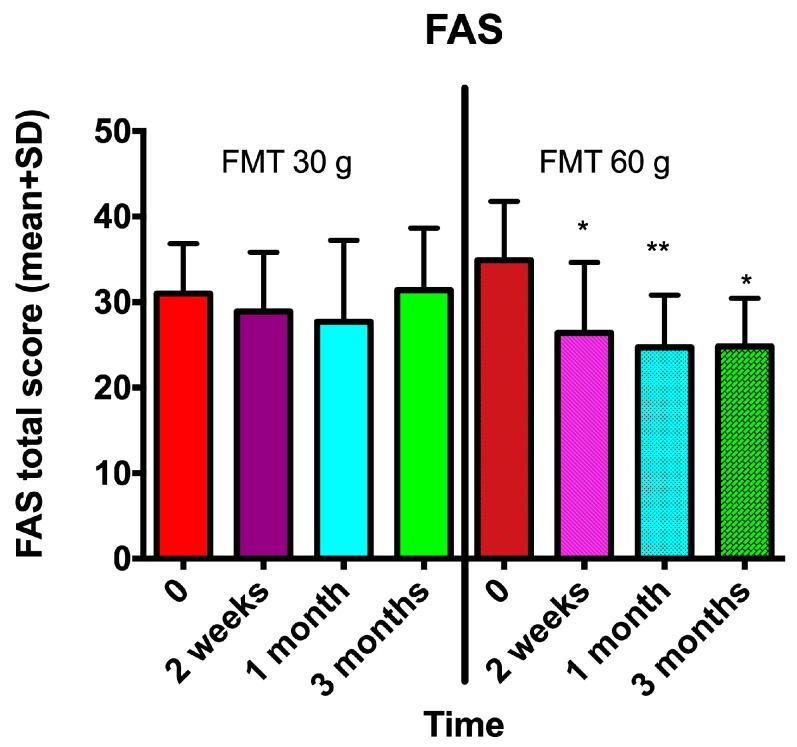
Fatigue as measured by the Fatigue Assessment Scale (FAS) questionnaire was reduced in IBS patients after they received a 60-g transplant but not when they received a 30-g transplant. * *p* < 0.05; ** *p* < 0.01 compared to baseline.

**Figure 4:**
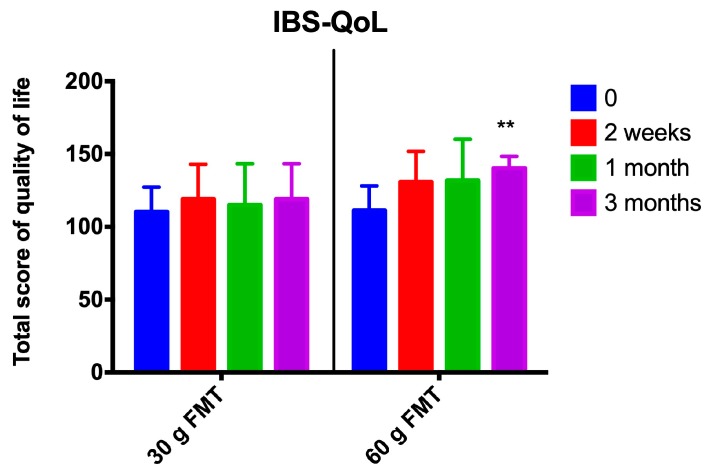
The quality of life as assessed by the IBS Quality of Life (IBS-QoL) questionnaire after FMT with 30-g and 60-g transplants. ** *p* < 0.01 compared to baseline.

**Figure 5:**
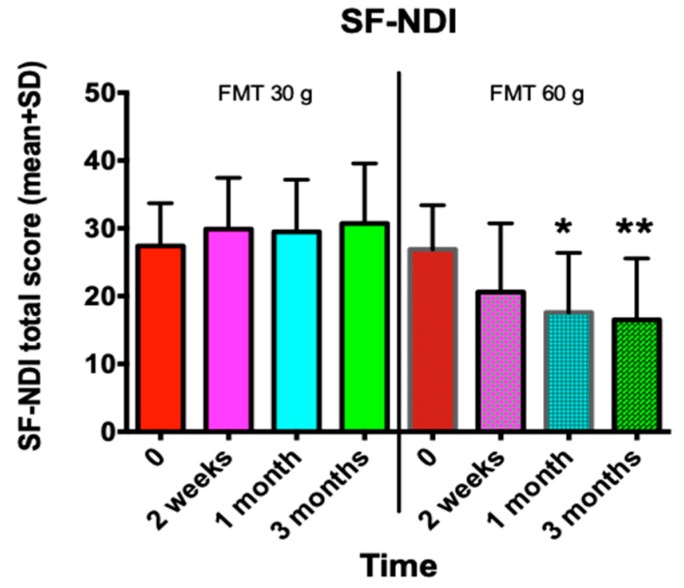
The change in the quality of life as measured by the Short-Form Dyspepsia Index (SF-NDI) questionnaire in IBS patients following FMT with 30-g and 60-g transplants. * *p* < 0.05; ** *p* < 0.01 compared to baseline.

The abdominal symptoms as assessed by the IBS-SSS and Birmingham IBS symptom questionnaires were significantly reduced following the FMT with the 60-g transplant (Figure 1; Figure 2), as was the fatigue score (Figure 3). The quality of life of the patients improved after they received the 60-g transplant, as indicated by their scores for the IBS-QoL and SF-NDI questionnaires (Figure 4 and Figure 5).

### 3.2. Dysbiosis Index

**Figure 6:**
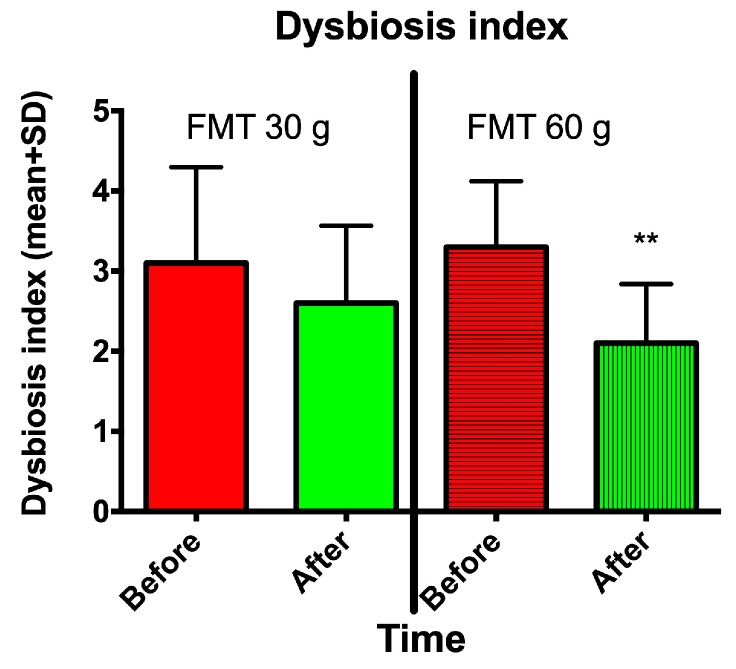
The dysbiosis index (DI) in IBS patients at baseline and 1 month after FMT with 30-g and 60-g transplants. ** *p* < 0.01 compared to baseline.

The DI decreased significantly following FMT with a 60-g transplant but not with a 30-g transplant (Figure 6).

## 4. Discussion

As mentioned previously, the intestinal bacterial profile of IBS patients differs from that of healthy subjects and shows a low diversity (dysbiosis) [4,5,6,7,8,9,10,11,12,13,14,15,16,17]. Among the important factors that determine the composition of gut microbiota are genetic factors, diet and drug intake, especially antibiotics [18]. Genetic factors explain only 5–10% of the intestinal bacterial composition [24]. Thus, environmental factors appear to be of great importance in determining the intestinal bacterial profile [24,36]. Despite the gene(s) responsible for IBS not being known yet, IBS seems to be hereditary [1,3] and this can at least partially explain the abnormalities in intestinal microbiota. Moreover, IBS patients avoid certain food items that they believe trigger their symptoms [37]. This might affect their intestinal bacterial profile. Furthermore, proton pump inhibitors (PIPs) have been reported to affect the intestinal bacterial composition [38]. In a recent study on a large cohort of IBS patients, 66% of the patients suffered from gastro-oesophageal reflux, 97% had endoscopic-verified erosive oesophagitis, and most of the patients used PIPs.

FMT appears to be effective at improving the symptoms and quality of life of patients with IBS, and also at reducing their fatigue [18,20,21,22,25]. The apparent efficacy of frozen faeces facilitates the use of FMT in the clinic by simplifying the logistical problems associated with FMT and makes it possible to establish faeces banks [22,25,39]. The outcome of FMT is dependent on the donor, and utilising a superdonor who is normobiotic and has a favourable specific bacterial signature is essential for success, although what constitutes a “favourable” bacterial signature remains somewhat unclear [24]. We recently identified that such a signature should include an abundance of *Streptococcus*, *Dorea*, and *Lactobacillus* spp. and bacteria belong to the Ruminococcaceae family. There are several issues that need to be clarified before FMT can be applied routinely to IBS patients in the clinic, such as the route of transplant administration, the transplant dose and whether the transplantation should involve a single or repeated dose.

FMT can be achieved by administering the transplant to the duodenum via a nose catheter or the working channel of a gastroscope, or to the colon via the working channel of a colonoscope or as an enema [26]. It has been shown that administering the transplant to the duodenum is effective [20,25]. This makes FMT easier to perform in the clinic, since a gastroscopy takes a shorter time to perform than a colonoscopy and does not require bowel preparation. Regarding the transplant dose needed for a successful FMT, we have shown recently that the response rate is higher and the effect occurs earlier and is more intense for a 60-g transplant than for a 30-g transplant.

The question addressed by the present study was whether the IBS patients who did not respond to a 30-g transplant in our previous study would respond to a 60-g transplant. The present findings showed that 70% of these patients responded to the FMT, and that increasing the dose of the transplant and/or repeating FMT improved their abdominal symptoms, fatigue, quality of life and dysbiosis.

The present study showed further that increasing the dose of the transplant and/or repeating FMT led to the complete remission of abdominal symptoms in 57% of the responders and to clinically significant improvements in fatigue and quality of life in 80% and 67% of the patients, respectively [27,31,33]. However, the present study had the following limitations: it investigated a small cohort of patients and did not study the long-term effects of FMT. Furthermore, the patients enrolled in the study adhered to a NICE-modified diet and therefore the outcomes cannot be extrapolated to the whole IBS population.

## 5. Conclusions

FMT is an effective treatment for patients with IBS. The success of FMT with frozen faeces administered via the upper gastrointestinal tract makes this treatment easy to perform in everyday clinical work. However, the availability of a superdonor and high-dose and/or repeating FMT are required.
